# Confirmation of Morphine Symptoms

**Published:** 1896-03

**Authors:** Thomas Skinner


### CONFIRMATION OF MORPHINE SYMPTOMS.

DEAR EDITOR:

I am indebted to THE HOMŒOPATHIC PHYSICIAN for the following most interesting case:

Mrs. B., a relative of my own by marriage, and whose constitution I fairly understand, consulted me on the 8th of January, 1896. For a couple of weeks she wrote me that she was suffering from giddiness, which is always worse after food or drink. As I know of no medicine better suited to this condition of the stomach and sensorium, I sent her Nux$^{1m}$ (F.C.), one powder to be dissolved in a teacup of cold water, and a teaspoonful to be taken before meals.

January 10th.—She writes me to say that the giddiness is better, or less, as regards meals and drinking, but that it is assuming a worse form, namely: "*I become unconscious and all becomes dark—worse by movement of any kind, especially of my head, and by eating or drinking.*"

On consulting my Repertories and the *Materia Medica*, the following medicines have giddiness on *moving* the head: Chin., Clem., Equis., Nat-m., Pip-m., and Samb.; *Rapidly*, Calc-c., Cb-v., Genist-t., Kal-bi., Mosch., Pho., and Sang-c.; *Turning head*, Agar., Amph., Cimex, Clem., Indm., Kal-c., Nat-c., Ptel., Rhus, Spig., Staph.; *Suddenly*, Lact-ac.; *Quickly*, CALC-C.

None of these satisfied me, as I knew her of old to be very nervous and a windy Lycopodium subject; and as Lyc. has well-marked giddiness when drinking, I sent her Lycopodium $^{2cm}$ (F. C.), one dose.

As I could not visit her, and as I knew she could not come to me alone, and knowing that a younger unmarried sister could accompany her, I begged of her to pay me a visit, which she did on the 13th of January.

Mrs. B. informed me that the single dose (Lyc.$^{2cm}_{!}$) had done her no good, and that instead, she thought that she was, if anything, worse, as when sitting at dinner her head or face had fallen into her plate of soup. The following photograph of her case is exact:

Vertigo from "*the least movement of her head*," if eating or drinking, with a tendency of the head to fall forwards, being perfectly unconscious for some little time. She has suffered in this way for two weeks, and worse lately, since taking the Lycop.$^{2cm}$.

I again prescribed Nux $^{m}$ as an antidote to the Lycop.$^{2cm}$, a dose at meals. Mrs. B. had left my consulting rooms (my office) about an hour when the December number of THE HOMŒOPATHIC PHYSICIAN arrived by post. On opening it, my surprise may be more easily imagined than described—the very page where it opened, almost of itself, page 563—I saw and read, "Vertigo, *from least motion of the head* (two days), a symptom of Morphine." I had no attenuated Morphia by me, but I had Morphia-mur.$^{500}$ (F.C.), so I made a powder of it and mailed it at once to Mrs. B.'s address, who resides about ten miles out of town, in Surrey. *Direction.*—Dissolve this powder in a teacup of cold water, and take one teaspoonful every four hours till *better* or worse. Be sure and let me know the effect.

January 15th.—"My Dear Dr. Skinner: I thank you very much for the medicine you so kindly sent me. I am thankful to be able to tell you that *it has done wonders for me.* I have taken six doses. The first two did not seem to have any effect. I felt just the same—rather worse, if anything different; but after the third dose I was considerably better, and since taking the sixth I have felt quite well." This powder of Muriate of Morphia $^{500}$ (F. C.) reached Mrs. B. before any of the Nux was taken, which may account for the aggravation of the first two doses. Alluding to the Lycop. $^{2cm}$, she says: "The medicine you gave me on Friday last did me no good. I fell down three times on Saturday and twice on Sunday. I cannot tell you what I have suffered with this dreadful feeling. It seems to have taken ten years from my life. The only thing that remains of my ailment, or which reminds me of it, are the numerous bruises, the result of my many tumbles. I may say that I am now *perfectly well!* And as my husband says that I am not *worse* tempered than usual, I presume I must be well!" In conclusion, Mrs. B. called to-day, by my request, to let me see how well she is. At the same time I wished to let her see the providential way in which her cure had been brought about.

On reading over to her Dr. Thurston's symptoms from one grain of Morphia, she recognized as corresponding to her own case, as follows: "Spare, tall" (Mrs. B. is six feet), "nervous temperament; dull pain in head; brain feels tense, as though wound up tightly; *vertigo from the least motion of head;* sudden attacks of fainting—several times a day." Add to this *unconsciousness, all becoming dark, and falling forward,* and we have a key-note to Morphia and Morphia-mur.

*Remarks.*—I may be wrong, but I am very much inclined to think that there is a phase of epilepsy in this case. The loss of consciousness, however temporary, and the falling forward are very suspicious. There were no convulsions of any kind. I have to thank you, Mr. Editor, and Dr. Rufus L. Thurston, of Boston, Mass., for enabling me to be of such signal benefit to my dear relative and patient, Mrs. B., while I remain

Yours faithfully,

THOS. SKINNER, M. D.

